# Seatbelts in CAR therapy: How Safe Are CARS?

**DOI:** 10.3390/ph8020230

**Published:** 2015-05-08

**Authors:** Kentaro Minagawa, Xiaoou Zhou, Shin Mineishi, Antonio Di Stasi

**Affiliations:** 1 Bone Marrow Transplantation and Cellular Therapy, University of Alabama at Birmingham, Birmingham, AL 35294-3300, USA; E-Mails: kminagawa@uabmc.edu (K.M.); smineishi@uabmc.edu (S.M.); 2 Center for Cell and Gene Therapy, Baylor College of Medicine, Houston, TX 77030-2399, USA; E-Mail: xxzhou@txch.org

**Keywords:** chimeric antigen receptor, suicide gene, safety switch, adoptive immunotherapy, cell therapy

## Abstract

T-cells genetically redirected with a chimeric antigen receptor (CAR) to recognize tumor antigens and kill tumor cells have been infused in several phase 1 clinical trials with success. Due to safety concerns related to on-target/off-tumor effects or cytokine release syndrome, however, strategies to prevent or abate serious adverse events are required. Pharmacologic therapies; suicide genes; or novel strategies to limit the cytotoxic effect only to malignant cells are under active investigations. In this review, we summarize results and toxicities of investigations employing CAR redirected T-cells, with a focus on published strategies to grant safety of this promising cellular application.

## 1. Introduction

Allogeneic hematopoietic cell transplantation (allo-HCT) is a potent form of immunotherapy [1], with anti-tumor effects orchestrated by both innate and adaptive immune system components. Donor lymphocytes infused with the graft or given post-transplant for leukemia relapse can mediate an anti-tumor effect, in virtue of recognition of tumor associated antigens (TAAs) and/or minor histocompatibility antigens (mHags) [2].

However, since most TAAs are aberrantly expressed self-proteins, resulting in T-cells with low-affinity T-cell-receptor (TCR), it is possible that the alloreactive component is more determinant for the graft-versus-tumor-effect (GVT). Although very effective in chronic myeloid leukemia, donor lymphocyte infusion (DLI) has proven to be of limited efficacy in acute leukemia, with less than 25% survival after 2 years [2].

Additionally, complications related to graft-versus-host-disease (GVHD) are responsible for the demise or low quality of life of a significant proportion of patients, as GVHD has been reported in up to 50% of patients receiving HCT from a matched donor. Although GVT in the absence of GVHD has been reported [3], there is overlap between the two mechanisms, because mHags, responsible of GVHD and GVT, can be expressed on both hematopoietic and non-hematopoietic tissues [4].

Clinical trials with mHag specific T-cells expanded and adoptively transferred to patients with disease relapse after transplantation have induced transient complete remissions (CR) in some patients [5], however, the strategy was logistically complex, and T-cells were expanded *in vitro* for up to 12 weeks. Since a long tissue culture period can result in T-cell exhaustion, this could be one potential culprit for their limited persistence in patients.

One alternative approach is to genetically redirect T-cells by endowing them with a transgenic TCR or chimeric antigen receptor (CAR). However TCR redirected T-cells are HLA restricted, and TCR mispairing with the endogenous TCR could result in reduced avidity or unwanted specificities [6]. Alternatively, CARs represent a universal platform for immune-therapy because they are not HLA-restricted, combining the specificity of an antibody with the killing machinery of the T-cell in a single chain [7], with a minimized risk of chain mispairing. Additionally, recognizing antigens in an HLA independent fashion makes CAR T-cells intrinsically resistant to immune evasion strategies that could arise during antigen processing or presentation.

Generally, CAR T-cells can only recognize surface molecules, which are often non-polymorphic and often shared between normal and tumor cells, raising justified concerns about their safety. As a matter of fact, infusion of CAR redirected T-cells has resulted in complete remission of disease in cases of refractory leukemia, but at the expense of frequent cytokine release syndrome [8,9,10,11,12,13,14,15,16,17,18,19], and even fatal on-target/off-tumor effects when targeting TAA in solid cancers [20]. These issues prompted the Recombinant DNA Advisory Committee of the National Institute of Health to draw some clinical recommendations, including implementing careful dose-escalation plans and co-expressing a suicide gene for switching-off unpredicted or controlling long-term toxicities [21].

In this review we will discuss modern concepts and applications on granting the safety of gene modified autologous or allogeneic T-cell applications for cancer immunotherapy.

## 2. CAR T-Cells in the Autologous Setting

**Figure 1:**
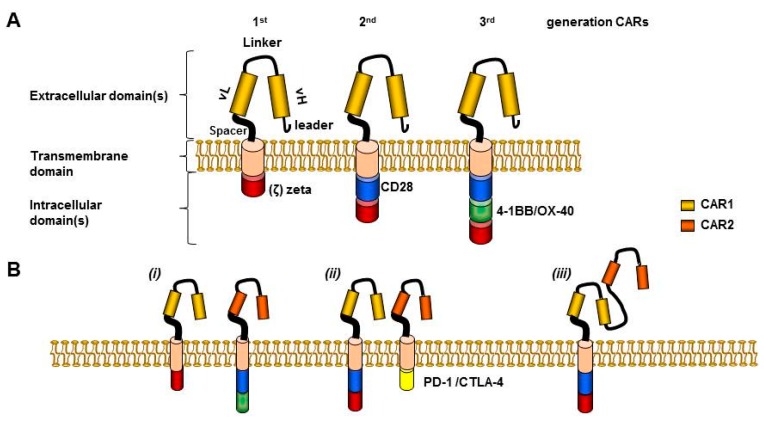
CAR constructs and dual targeting pre-clinical approaches to reduce toxicity. **(A)** CAR extracellular domain includes leader sequence, single chain variable fragment (scFv) (H (heavy) and L (light) chain), connected by a linker, (e.g., SG..GS). A spacer, (e.g., hinge region of human immunoglobulin D molecule) grants flexibility and joins to the transmembrane domain (TM). TM includes for example the constant region of the human G immunoglobulin, whereas the intracellular domain includes CD3 zeta endo-domain (1st generation CARs), plus minus a CD28 domain (2nd generation), or plus minus an additional co-stimulatory domain (3rd generation CARs) [28]. **(B)** “Dual targeting” approaches to reduce toxicities: (i) Trans-signaling CAR divides killing domain and co-stimulatory domain in separate molecules targeting two distinct tumor antigens, limiting CAR activation capacity when only a single antigen is engaged [29,30,31]. (ii) Inhibitory CAR replaces the zeta chain with an inhibitory domain, overcoming the activatory signal from a coexpressed activatory CAR [6]. (iii) Tandem CAR is composed by two different antigen binding fragments allowing targeting of two different antigens by a single construct, with synergistic effect when both are engaged simultaneously [32]. CAR-1 and CAR-2 used as example, recognized antigen 1 and antigen 2, respectively.

Considering that first generation CARs (Figure 1A) had limited expansion and persistence [22,23,24,25], investigators engrafted a CAR onto the surface of virus-specific T-cells in order to exploit the co-stimulation provided by antigen-presenting cells cross-presenting viral antigens. Eleven children affected by neuroblastoma with active disease were given Epstein–Barr-virus (EBV) specific cytotoxic-T-lymphocytes modified with a first generation CAR redirected towards the disialoganglioside GD2. Gene modified cells persisted for weeks after *in vivo* transfer and mediated objective responses in almost half of the cases, with three out of eleven patients achieving complete remission (CR) [26,27].

In a further attempt to improve expansion and persistence of CAR T-cells investigators added one or more co-stimulation endodomains in frame with the zeta chain, and thus second and third generation CARs have been generated, respectively, primarily enhancing CAR mediated proliferation and protecting T-cells from activation-induced cell death. (Figure 1A). Indeed, these attempts proved successful in preclinical models [33,34,35,36,37], and later entered clinical trials confirming improved expansion and persistence in patients with CD19^+^ lymphoid malignancies who received second generation CAR-CD19 T-cells as compared with co-infused first generation CAR-CD19 T-cells [25].

In clinical trials from several institutions targeting relapsed/refractory ALL impressive clinical results have been reported [8,9,10,11,12,13,14,15,16,18,25]. All in all, CAR-CD19 T-cells for ALL have been reported to control the disease and induce remission in patients with relapsed/refractory disease. The majority of trials included lymphodepleting chemotherapy in an effort to create a microenvironment favorable for homeostatic T-cell expansion.

Davila *et al.* infused CAR T-cells with a CD28 costimulatory domain and reported that 50% of adult ALL patients became eligible for stem cell transplantation, remaining in remission thereafter at the last follow-up [14]. Maude *et al.* [15], reported on 30 children and adults receiving CAR-CD19 T-cells with 4-1BB costimulation domain, and CR was achieved in 27 patients (90%), including two patients with blinatumomab-refractory disease and 15 patients who had undergone stem-cell transplantation previously. CAR T-cells proliferated *in vivo* and were detectable in the blood, bone marrow, and cerebrospinal fluid of patients who had a response. The 6-month event-free survival rate was 67%, with an overall survival rate of 78%. The authors demonstrated in this trial that ongoing remission for up to 2 years is possible with CAR therapy even in the absence of allo-HCT.

Lee *et al.* [18], enrolled children and young adults mainly with relapsed or refractory ALL. Autologous T-cells were engineered to express a CD19-CAR incorporating the CD28 signaling domains. CD19-CAR therapy induced a CR in 70% of patients with B-ALL and an MRD-negative complete response in 60%. Ten of twelve patients who became molecular residual disease (MRD)-negative went on to receive an allo-HCT remaining disease free at a median follow-up 10 of months.

Some successes have been recorded also with CAR-CD19 in refractory chronic lymphoid leukemia (CLL) patients [8,13], whereas larger studies on CAR-CD19 are needed in non-Hodgkin lymphoma [17,18,25,38] with some preliminary encouraging results using CAR-CD20 T-cells [39,40]. Additional modifications may be necessary for optimal efficacy of CAR T-cell therapies in solid tumors [22,23,24,41,42], although some evidence of tumor regression has been seen in patients with advanced solid cancers receiving mRNA anti-mesothelin electroporated T-cells (RNA-CAR-T-meso cells) [43,44,45,46], whilst CRs of disease were seen in patients with neuroblastoma with GD2 redirected CAR T-cells therapy [26,27]. The list of antigens targeted by CARs is rapidly extending, as nicely reviewed elsewhere [2].

Active investigations are attempting to improve the preservation of effector function, self-renewal, engraftment, and homing abilities of *ex vivo* expanded genetically redirected T-cells. To note, in the majority of these studies T-cells were activated with CD3/CD28-beads rather than soluble anti-CD3 antibody, which might have contributed to the improved efficacy *in vivo*.

It will need to be assessed in clinical trials whether the transduction of effector T-cells generated from naïve [47,48], or central memory subsets [49], will result in enhanced expansion, persistence and anti-tumor activity in patients. The Italian group showed that the activation of naïve T-cells with anti-CD3 and anti-CD28 antibody-conjugated beads in the presence of low dose interleukin (IL)-7 and IL-15 promoted the generation of T-stem-cell-memory cells (Tscm) able to persist, expand, and differentiate across serial transplants in mice, suggesting self-renewal abilities. Interestingly, the increased efficacy of naive-derived Tscm cells *in vivo* strictly depended on them being purified prior to *ex vivo* manipulation, as naive T-cells expanded in the presence of other subsets failed to engraft upon serial transplants [50].

## 3. Side Effects from CAR T-Cells Infused in the Autologous Setting

CAR T-cells have resulted in on-target/off-tumor effects and cytokine release syndrome (CRS) in clinical trials, as well as in significant myelosuppression in preclinical models of acute myeloid leukemia (AML).

Serious adverse events (SAEs) can be related to several factors, including the infused cell dose, the presence of residual morphological disease in the recipient, and the magnitude and intensity of expression of the targeted antigen on normal tissues.

Since on-target/off-tumor effects are due to the fact that the TAA is shared on normal tissue, in order to minimize the risk of toxicity by genetically redirected T-cells, the first step would be to select antigen with preferential expression on tumor cells. However, since such antigens are not always available, *in vitro* and/or *in vivo* assays are performed in an attempt to predict the risk of toxicity when targeting a certain antigen. In *vitro* cytotoxic assays challenging TAA redirected T-cells with target tumor cells expressing the antigen of interest, including cells derived from normal tissues might not reproduce the tridimensional complex *in vivo* matrix.

*In vivo* animal models involve humanized antigen transgenic mice involving the infusion of human genetically redirected T-cells in mice expressing human antigen(s), or surrogate mouse models which entail engrafting a CAR molecule onto murine T-cells targeting the relevant antigens but of murine origin. Although, CRS or B-cell aplasia have been studied in animal models [51,52], there are several limitations that may limit the reproducibility of these observation in humans, such as the potential different correlation between bioequivalence and bio distribution between animals and humans, as well as differences in antigen expression, or in the expression of co-stimulatory molecules and/or in the cytokine milieu. The use of non-human primates models has been also employed to evaluate the toxicity of genetically redirected T-cells towards normal tissues, with the advantage of displaying a superior degree of homology with human protein sequences [53].

As anticipated earlier, in an effort to increase *in vivo* expansion and anti-tumor activity, second/third generation CARs have been developed. However, it is anticipated that the infusion of second/third generation CAR T-cells might associate with an increased risk of toxicity, due to superior T-cells activation, and/or co-stimulation. Indeed, infusion of CAR T-cells endowed with a second generation CAR has resulted in a high incidence of severe CRS (sCRS). In solid tumors third generation CAR T-cells redirected toward the HER-2 antigen caused fatal lung toxicity due to low level expression of HER-2 on lung epithelia combined with the high potency of the CAR containing the co-stimulatory molecules CD28 and 4-1BB [20].

B-cell aplasia and/or CRS have been frequently reported from CAR-CD19 T-cell studies [8,9,10,11,12,13,14,15,16,17,18,19]. With some patients developing alarming but reversible neurological symptoms including delirium and seizure-like activity, the latter likely related to generalized T-cell mediated inflammation rather than direct toxicity of CAR T-cells in the brain. CAR T-cells applications for myeloid leukemia have been tested in murine models, and further clinical development has been hampered by the evidence of significant myelosuppression [54,55,56,57], and therefore strategies to overcome this SAE need to be implemented, such as the development of strategies to spare normal hematopoietic stem cells and mature hematopoietic progenitor cells [58].

One other potential SAE that could manifest when using cells modified with integrating vectors is insertion in hot-spots resulting in oncogene de-regulation with malignant transformation. Although this remains a concern when transduced stem cells have been infused [59], this SAE has never been reported to date in clinical studies employing transgenic T-cells [60].

## 4. Recommendations from the Recombinant DNA Advisory Committee of the National Institute of Health

Those recommendations [21] were compiled to provide guidance to the investigators of CAR T-cells clinical studies. The recommendation differentiate between clinical trials based on the infusion of first generation CAR redirected T-cells (excluding EBV-specific cytotoxic-T-cells), *versus* clinical trials based on the infusion of second/third generation CAR redirected T-cells, providing guidance on dosing, administration of cytokine or preconditioning treatment, as well as proposing dose infusion schedules. Suggested initial doses for clinical infusion are 3 × 10^6^/kg, 3 × 10^6^/kg, 3 × 10^5^/kg for first generation CAR redirected T-cells, first generation CAR-engrafted on EBV T-cells, or second/third generation CAR redirected T-cells, respectively. The guidelines recommends reducing the afore mentioned doses down to 3 × 10^4^/kg, 1 × 10^4^/kg, and 1 × 10^4^/kg, respectively, when targeting novel antigens. It needs to be kept in mind that these suggested dosings are based on the transfer of unselected CAR T-cells, whereas eventual prior subset selection may affect toxicity.

Regarding the use of cytokines support, while this can be useful in improving expansion of first generation CAR, its potential benefit needs still to be explored in more depth when using second/third generation CAR T-cells. Conversely, the use of preconditioning chemotherapy it is regarded as potentially useful to enhance the survival and the engraftment of infused second/third generation CAR T-cells.

Additionally, in order to reduce the risk of toxicity from the infused cells, a split dose infusion strategy in second/third generation CAR T-cells is warranted, such as to administer 30% of the dose on day 1 and 70% on day 2 (after adequate safety assessment), or to administer them in 10%, 30%, and 60% fractionated schedule over three days, for example.

Finally, the co-expression of a suicide gene is also recommended for switching-off unpredicted or controlling long-term toxicities, although is unknown if they would be able to attenuate SAEs once they become clinically evident [21].

## 5. Therapeutic Approach to Cytokine Release Syndrome

CRS is as an inflammatory response clinically manifesting with fever, nausea, headache, tachycardia, hypotension, hypoxia, as well as cardiac, or neurologic manifestations. Diagnostic criteria include: (i) fever for at least three consecutive days, (ii) two cytokines max fold change of at least 75 or one cytokine max fold change of at least 250, (iii) at least one clinical sign of toxicity such as –hypotension (requiring at least one intravenous vasoactive pressure) or –hypoxia (PO2 < 90%), or –neurologic disorders (including mental status change, obtundation and seizures) [14].

Although some side effects, such as the B-cell aplasia resulting from CAR T-cells targeting B-cell associated antigens can be easily managed with gamma globulin replacement and long term with an allo-HCT (which is the standard of care for eligible patients), no standardized treatment exists for CRS, which has been managed to date with steroids, or more recently with the interleukin-6 monoclonal antibody tocilizumab.

A sCRS treatment algorithm has been recently proposed [14]. This algorithm poses a particular alert to patients with morphological residual disease receiving CAR T-cells for the higher risk of sCRS, likely in virtue of interleukin-6, production by monocytes/macrophages after phagocytosis of tumor debris, (macrophage activation syndrome, or MAS). Also, since it was observed that only patients with sCRS had a C reactive protein (CRP) level of more or equal 20 mg/dL, this was included to guide decision making as a surrogate for cytokine elevation/inflammation [14].

The proposed algorithm can be exemplified in a four steps approach: (1) if the patient would develop fever for at least two days the initiation of levetiracetam is proposed; (2) in case of CRP levels more or equal 20 mg/dL the recommendation for intensive care unit (ICU)-transfer is given; (3) in the presence of clinical signs of toxicity, the algorithm suggests to consider the administration of tocilizumab; (4) if the patient’s clinical status fails to improve after a second dose of tocilizumab is given, the administration of steroids is warranted, such as dexamethasone 10 mg intravenously (I.V.) twice daily. Although these guidelines are awaiting prospective validation, they represent a valid example-tool to use as guidance in the systematic management of patients with CRS. However, since tocilizumab or corticosteroids can also abrogate the anti tumor efficacy of the infused T-cells, strategies aiming at the selective elimination of allo-reactive T-cells, (and/or ameliorating the inflammatory process without complete abrogation of the infused cells) needs to be investigated and/or implemented.

## 6. Strategies to Ensure Safety

Considering the SAE manifested after the infusion of CAR T-cells, it is desirable to implement strategies to alleviate and/or abate toxicity. Since main part of toxicity resulting from T-cell infusion is related to expansion of T-cells, and cannot be reduced by stopping the offending agent as with pharmacologic agents, strategies such as the incorporation of a suicide gene or the development of approaches aiming at selectively redirecting T-cells to selectively kill tumor cells, hold promise in reversing untoward effects.

### 6.1. Suicide Gene Applications

A suicide gene is a genetically encoded molecule that allows selective destruction of adoptively transferred cells. Suicide gene addition to cellular therapeutic products can lead to selective ablation of gene-modified cells, preventing collateral damage to contiguous cells and/or tissues.

Suicide gene technologies can be broadly classified based upon their mechanism of action into three categories, metabolic [61,62,63], dimerization inducing [64,65], and safety switches mediated by therapeutic monoclonal antibody [66,67,68,69,70]. Table 1 presents an overview of the most investigated suicide gene technologies to date.

The ideal agent for suicide gene activation should be biologically inert, have an adequate bio-availability and bio-distribution profiles, and be characterized by acceptable or absent intrinsic toxicity.

**Table 1:** Classification and main characteristics of suicide gene technologies.

Mechanism of Action (Source)	Activating Agent	Mechanism of Action	Percentage of Transduced Cell Elimination in Patients	Advantages	Drawback(s)	Refs.
Metabolic (viral) e.g., HSV-TK	Ganciclovir	-Ganciclovir triphosphate mediated Interference of DNA synthesis; -Apoptosis through CD95 aggregation	NR, *in vivo* depletion of alloreactive cells	-Gradual onset -Eliminates alloreactive cells when used in allo setting	-Preclude therapeutic use of ganciclovir -Immunogenic	[61,71,72]
Dimerization inducing (human) e.g., iCasp9	Non-therapeutic small molecule dimerizer	-iCasp9 dimerization and activation of downstream caspases resulting in apoptosis	Incomplete, but >=90% with *in vivo* depletion of alloreactive cells	-Rapid onset -Eliminates alloreactive cells, and non-immunogenic when used in allo setting -Uses non-therapeutic agent	-Kills ≥90% of cells -Uses non commercially available dimerizer	[64,73]
Therapeutic mAb mediated (human) e.g., CD20	mAb	-Antibody/complement dependent cellular cytotoxicity	Not done	-Rapid onset -Non-immunogenic when used in allo setting	On-target toxicity from each specific mAb used needs to be considered	[66,67,68,69,70,74]

HSV-TK: herpes-simplex-virus thymidine-kinase, NR: not reported, allo: allogeneic, iCasp9; inducible Caspase9, mAb: monoclonal antibody.

A suicide gene strategy should be designed for each specific application, taking into account the nature of target cells, the source of the suicide gene, the type of activating agent, the onset of action, and the elimination’s kinetic of the target population.

Metabolic suicide genes are based on the conversion of a nontoxic drug into a toxic compound in gene-modified cells. One such example, herpes simplex virus thymidine kinase (HSV-TK) which phosphorylates nucleoside analogs, including ganciclovir (GCV), and its resulting triphosphate form incorporates into DNA via the action of DNA polymerase, leading to chain termination and cell death [75]. HSV-TK/GCV also induces apoptosis through CD95-L independent CD95 aggregation, leading to the formation of a Fas-associated death domain protein (FADD) and caspase-8-containing death-inducing signaling complex [76].

Apoptotic genes (e.g., *caspases*) eliminate cells by inducing apoptosis [64,73] The *iCasp9* suicide gene [65,77] consist of *FKBP12-F36V* domain linked, via a flexible *Ser-Gly-Gly-Gly-Ser* linker to *∆Caspase 9*, which is caspase without the recruitment domain. *FKBP12-F36V* consists of a *FKBP* domain with a substitution at residue 36 of phenylalanine for valine, binding synthetic dimeric ligands, such as the otherwise inert small molecule AP1903 [78], with high selectivity and subnanomolar affinity. The transgenic cassette also include a truncated CD19 (*ΔCD19*) molecule, serving as a selectable marker to ensure ≥90% purity.

Suicide gene safety and effectiveness has been shown in clinical trials using HSV-TK or iCasp9 suicide gene modified DLI, resulting in abrogation of both acute [62,64,71,79,80] and chronic GVHD [72].

HSV-TK suicide gene modified DLI has also exerted a GVT effect [62,71,79,80], even in patients in whom antibodies against HSV-TK developed. One explanation could be that the gradual elimination of HSV-TK contributed to a protracted GVT effect [81]. Additional indirect evidence suggesting a GVT effect comes from finding *de novo* loss of the mismatched HLA expression on leukemic blasts in one patient at the time of relapse [80]. Larger studies and longer follow-up are needed to define the GVT effect of iCasp9 DLI.

Interestingly, in suicide gene DLI clinical trials long-term persistence of residual non-alloreactive T-cells was observed. One possible explanation is the preferential elimination of cells actively proliferating during GVHD in virtue of their higher transgene expression with sparing viral and fungal reactive T-cells [73]. While this can represent an advantage when used for DLI, complete elimination of CAR/TCR redirected T-cells or hematopoietic stem cells may be necessary for the SAE to abate. For this reason it is important to ensure that the majority (>99%) of the infuse cells would express the suicide gene, for example through cell sorting, in order to ensure maximal cell elimination.

Pre-clinical experiments expressing the iCasp9 in conjunction with CAR-CD19/CD20 T-cells have proven the feasibility of such an approach [82,83], and phase 1 clinical trials in patients with sarcoma or neuroblastoma receiving iCasp9 T-cells co-expressing a CAR against the disialoganglioside GD2 molecule (Clinicaltrials.gov identifier NCT01822652 and NCT01953900, respectively) are ongoing. If toxicity is related to the transduced T-cells only, selectable markers could be obviated, especially in an autologous setting, provided that all the transduced cells also harbor the suicide gene, in order to be eliminated in case of SAEs.

Several strategies can be employed to ensure that all CAR T-cells harbor the suicide gene. Cells may be transduced with a bicistronic vector with the suicide gene cloned before the CAR construct to ensure all CAR bearing cells also express the suicide gene. Alternatively a selectable marker could be introduced enabling sorting and enrichment for suicide gene expressing cells, and this strategy would be strongly recommended when using CAR T-cells in the allogeneic setting. Genetic modification of cells with a protein expressed in the plasma membrane, can enable selection, *in vivo* tracking and cell removal after administration of a specific monoclonal antibody. For example, retroviral delivery of the CD20 molecule into T-cells and anti-CD20 monoclonal antibody treatment post T-cell infusion has been validated in preclinical models as a suicide gene strategy [66,67,68]. As an extension of this concept, other interesting pre-clinical models have been investigated: an interesting application from Kieback *et al.* consisted in the introduction of a 10 amino acid tag of c-myc protein into the TCR sequence allowing tracking, selection, and elimination of the transduced cells after monoclonal antibody administration in a preclinical model, and this strategy could also be applied to CAR constructs [70]. Additionally, investigators from London generated a novel compact suicide gene (RQR8) combining epitopes from CD34 and CD20 enabling CD34 selection, cell tracking, as well as deletion after anti-CD20 monoclonal antibody administration [74]. Finally, another approach has used truncated human epidermal growth factor receptor (EGFR) polypeptide/anti-EGFR monoclonal antibody for selection, tracking and/or transduced cell elimination with the specific monoclonal anti-EGFR antibody [69].

### 6.2. Dual Targeting Strategies to Ensure Safety

Considering that suicide gene activation would eliminate the majority of CAR modified T-cells, it would be advisable to combine them with strategies able to mitigate side effects from infusion of transgenic T-cells, thus activating the suicide gene only as a last resort. However, the novel strategies explained below are yet to be investigated in the clinical setting.

One interesting approach to reduce untoward manifestations of CAR- T-cell therapy has been to modify the T-cells with two different CARs, each directed towards a different TAA epitope. In details, CAR No.1 would transmit the killing signal, whereas CAR No.2 would transmit the co-stimulation signal. This strategy would allow superior T-cell activation and effector function only when both antigens are engaged simultaneously [29,30,31] (Figure 1B*(i)*).

The goal of avoiding targeting normal tissues which share TAAs, has led researchers to devise a strategy aiming at co-expressing an ‘inhibitory’ CAR (iCAR). iCARs see the incorporation of cytotoxic T-lymphocyte-associated antigen 4 (CTLA-4) or programmed death-1 (PD-1) signaling domain, in spite of the zeta chain signaling domain, therefore transmitting an inhibitory signal, prevailing over the activating signal of the zeta chain resulting in sparing of the target (Figure 1B*(ii)*)*.* The iCAR would recognize a second antigen co-expressed on normal tissues together with the TAA targeted by the ‘activatory’ CAR. Importantly, T-cells would regain the ability to kill the target after disengagement of the iCAR. Fedorov *et al.* demonstrated feasibility and efficacy of this approach in a preclinical model [58].

Another novel approach, the “TanCAR” technology is aiming at targeting two different antigens, but using a single transgene construct. TanCAR consisted of two different antigen binding domains separated by a flexible linker [32], and the simultaneous engagement of both domains by two TAAs resulted in synergistic effect (*Figure 1**B(iii)*)*.* Importantly, even the engagement of a single domain by one TAA resulted in T-cell activation and killing of the target. Additionally, the use of T-cells electroporated with mRNA encoding a CAR molecule has been successfully validated in pre-clinical models and in one clinical trial. CAR expression and function of RNA-electroporated T-cells could be detected up to a week post electroporation, and multiple injections of RNA-CAR-T-meso-cells mediated regression of large vascularized flank mesothelioma tumors in mice, including engrafted autologous mesothelioma tumor cells isolated from the patient [46]. By granting transient CAR expression, this strategy could potentially offer a safer approach to limit the SAEs. The authors have demonstrated the safety and feasibility of this approach after I.V. infusion in two patients, one with advanced pleural mesothelioma and one with metastatic pancreatic carcinoma, with migration to primary and metastatic tumor sites resulting in reduced tumor burden, and decline in CA19.9 marker, respectively. Laboratory evidence of antitumor activity, and the RNA-CAR-T-meso cells elicited an antitumor immune response revealed by the development of novel anti-tumor antibodies were also observed [44]. As of concern with this strategy, the need for repeated infusions for optimal anti-tumor effect has resulted in anaphylaxis due to the development of an IgE immune response directed against the murine derived CAR [45]. However, the use of humanized scFv binding domains may reduce this risk, as well as short term persistence of the infused cells from a host mounted immune-response.

## 7. CAR T-Cells in the Allogeneic Setting

Although allo-HCT is a potentially curative strategy for patients with hematologic and lymphoid malignancies at high risk of relapse [1], additional strategies are needed to further reduce the risk of disease relapse after transplant [84]. One of the strategies to achieve this goal could be the prophylactic infusion of CAR redirected T-cells post-transplant, although one concern may be the absence of antigen in order to provide T-cell stimulation to grant expansion and persistence of the infused CAR redirected T-cells. Additionally, since it was reported that some cases of AML relapse after allo-HCT because of the selective loss of the mismatched HLA alleles as a result of the alloreactive pressure [80], these patients are exposed to the dreadful complications of alloreactivity, namely GVHD, without benefiting from its therapeutic effects. Therefore treatment with allogeneic CAR T-cells could help bypassing this immune-evasion strategy adopted by the tumor.

Ten patients with lymphoid malignancies who relapsed after allo-HCT received infusions of allogeneic CD19 specific CAR T-cells from the original donors with one patients experiencing CR (ongoing at nine months after T-cell infusion) without GVHD, although grade 3-4 toxicities were seen in four patients [19].

Allogeneic CAR T-cells could also result in GVHD, however, and therefore there is an even more stringent need to implement safety measures, ideally a suicide gene, because it currently represents the only effective safety measure validated in clinical trials of unmodified DLI.

Since suicide gene activation would also coincidentally eliminate the GVT effect, it would be ideal to switch the suicide gene on only if absolutely necessary.

Alternative strategies to minimizing the risk of GVHD other than suicide gene modification may include the strategies summarized in Figure 1B, or to perform CAR modification of T-cells that are naturally non-alloreactive, such as allodepleted T-cells [85], virus-specific T-cells [86,87,88], natural-killer T-cells [89], or gamma-delta T-cells [90]. In fact, for this reason authors have investigated a strategy to engraft a CAR-CD19 construct on the surface of gamma-delta T-cells with success, *in vitro* and *in vivo* in mice [91].

Regarding CAR modification of virus-specific T-cells (non-alloreactive), for example, a recent clinical trial involved CAR-CD19 engrafted onto the surface of T-cells specific for cytomegalovirus, adenovirus, or Epstein-Barr virus antigens. This trial enrolled patients with relapsed (N:6) or at high-risk of relapse (N:2) lymphoid malignancy following allo-HCT (N:2) [88], Objective responses, as assessed 6 weeks after T-cell infusion, were seen in two patients with relapsed disease after transplant, with two additional patients remaining in continuous CR, without accompanying GVHD in any patient. It is interesting to consider the attempt to reduce the risk of GVHD by making donor T-cells artificially non-alloreactive by knocking down the endogenous TCR, using genetic editing approaches [92].

## 8. Conclusions

Immunotherapy approaches for cancer treatment represent a potent tool to harness the GVT effect either in the autologous or the allogeneic setting. This is an exciting time, where progresses from gene therapy and immunotherapy are being recorded at an accelerated pace, with growing enthusiasm in both the scientific and the lay communities.

The years to come will see if immunotherapy approaches will hold their promise to replace or enhance standard pharmacologic anti-cancer therapies, as well as grant long-term anti-cancer surveillance, including in sanctuary sites, even when used as adjuvant therapy. Because the benefits need to overcome the risks, rigorous clinical validation of strategies to prevent or abrogate toxicities is warranted.

## References

[1] Copelan E.A. (2006). Hematopoietic stem-cell transplantation. New Engl. J. Med..

[2] Rambaldi A., Biagi E., Bonini C., Biondi A., Introna M. (2015). Cell-based strategies to manage leukemia relapse: Efficacy and feasibility of immunotherapy approaches. Leukemia.

[3] Ringden O., Labopin M., Gorin N.C., Schmitz N., Schaefer U.W., Prentice H.G., Bergmann L., Jouet J.P., Mandelli F., Blaise D. (2000). Is there a graft-versus-leukaemia effect in the absence of graft-versus-host disease in patients undergoing bone marrow transplantation for acute leukaemia?. Br. J. Haematol..

[4] De Bueger M., Bakker A., Van Rood J.J., Van der Woude F., Goulmy E. (1992). Tissue distribution of human minor histocompatibility antigens. Ubiquitous *versus* restricted tissue distribution indicates heterogeneity among human cytotoxic t lymphocyte-defined non-mhc antigens. J. Immunol..

[5] Warren E.H., Fujii N., Akatsuka Y., Chaney C.N., Mito J.K., Loeb K.R., Gooley T.A., Brown M.L., Koo K.K., Rosinski K.V. (2010). Therapy of relapsed leukemia after allogeneic hematopoietic cell transplantation with T cells specific for minor histocompatibility antigens. Blood.

[6] Thomas S., Stauss H.J., Morris E.C. (2010). Molecular immunology lessons from therapeutic T-cell receptor gene transfer. Immunology.

[7] Eshhar Z., Waks T., Gross G., Schindler D.G. (1993). Specific activation and targeting of cytotoxic lymphocytes through chimeric single chains consisting of antibody-binding domains and the gamma or zeta subunits of the immunoglobulin and t-cell receptors. Proc. Natl. Acad. Sci. USA.

[8] Kalos M., Levine B.L., Porter D.L., Katz S., Grupp S.A., Bagg A., June C.H. (2011). T cells with chimeric antigen receptors have potent antitumor effects and can establish memory in patients with advanced leukemia. Sci. Transl. Med..

[9] Grupp S.A., Kalos M., Barrett D., Aplenc R., Porter D.L., Rheingold S.R., Teachey D.T., Chew A., Hauck B., Wright J.F. (2013). Chimeric antigen receptor-modified T cells for acute lymphoid leukemia. New Engl. J. Med..

[10] Brentjens R.J., Davila M.L., Riviere I., Park J., Wang X., Cowell L.G., Bartido S., Stefanski J., Taylor C., Olszewska M. (2013). Cd19-targeted T cells rapidly induce molecular remissions in adults with chemotherapy-refractory acute lymphoblastic leukemia. Sci. Transl. Med..

[11] Brentjens R.J., Riviere I., Park J.H., Davila M.L., Wang X., Stefanski J., Taylor C., Yeh R., Bartido S., Borquez-Ojeda O. (2011). Safety and persistence of adoptively transferred autologous cd19-targeted T cells in patients with relapsed or chemotherapy refractory B-cell leukemias. Blood.

[12] Kochenderfer J.N., Wilson W.H., Janik J.E., Dudley M.E., Stetler-Stevenson M., Feldman S.A., Maric I., Raffeld M., Nathan D.A., Lanier B.J. (2010). Eradication of b-lineage cells and regression of lymphoma in a patient treated with autologous T cells genetically engineered to recognize CD19. Blood.

[13] Porter D.L., Levine B.L., Kalos M., Bagg A., June C.H. (2011). Chimeric antigen receptor-modified T cells in chronic lymphoid leukemia. New Engl. J. Med..

[14] Davila M.L., Riviere I., Wang X., Bartido S., Park J., Curran K., Chung S.S., Stefanski J., Borquez-Ojeda O., Olszewska M. (2014). Efficacy and toxicity management of 19–28z car t cell therapy in b cell acute lymphoblastic leukemia. Sci. Transl. Med..

[15] Maude S.L., Frey N., Shaw P.A., Aplenc R., Barrett D.M., Bunin N.J., Chew A., Gonzalez V.E., Zheng Z., Lacey S.F. (2014). Chimeric antigen receptor T cells for sustained remissions in leukemia. New Engl. J. Med..

[16] Kochenderfer J.N., Dudley M.E., Feldman S.A., Wilson W.H., Spaner D.E., Maric I., Stetler-Stevenson M., Phan G.Q., Hughes M.S., Sherry R.M. (2012). B-cell depletion and remissions of malignancy along with cytokine-associated toxicity in a clinical trial of anti-CD19 chimeric-antigen-receptor-transduced T cells. Blood.

[17] Kochenderfer J.N., Dudley M.E., Kassim S.H., Somerville R.P., Carpenter R.O., Stetler-Stevenson M., Yang J.C., Phan G.Q., Hughes M.S., Sherry R.M. (2015). Chemotherapy-refractory diffuse large B-cell lymphoma and indolent B-cell malignancies can be effectively treated with autologous T cells expressing an anti-cd19 chimeric antigen receptor. J. Clin. Oncol..

[18] Lee D.W., Kochenderfer J.N., Stetler-Stevenson M., Cui Y.K., Delbrook C., Feldman S.A., Fry T.J., Orentas R., Sabatino M., Shah N.N. (2015). T cells expressing cd19 chimeric antigen receptors for acute lymphoblastic leukaemia in children and young adults: A phase 1 dose-escalation trial. Lancet.

[19] Kochenderfer J.N., Rosenberg S.A. (2013). Treating b-cell cancer with T cells expressing anti-cd19 chimeric antigen receptors. Nat. Rev. Clin. Oncol..

[20] Morgan R.A., Yang J.C., Kitano M., Dudley M.E., Laurencot C.M., Rosenberg S.A. (2010). Case report of a serious adverse event following the administration of T cells transduced with a chimeric antigen receptor recognizing erbb2. Mol. Ther..

[21] Ertl H.C.J., Zaia J., Rosenberg S.A., June C.H., Dotti G., Kahn J., Cooper L.J.N., Corrigan-Curay J., Strome S.E. (2011). Considerations for the clinical application of chimeric antigen receptor T cells: Observations from a recombinant DNA advisory committee symposium held June 15, 2010. Cancer Res..

[22] Park J.R., Digiusto D.L., Slovak M., Wright C., Naranjo A., Wagner J., Meechoovet H.B., Bautista C., Chang W.C., Ostberg J.R. (2007). Adoptive transfer of chimeric antigen receptor re-directed cytolytic t lymphocyte clones in patients with neuroblastoma. Mol. Ther..

[23] Kershaw M.H., Westwood J.A., Parker L.L., Wang G., Eshhar Z., Mavroukakis S.A., White D.E., Wunderlich J.R., Canevari S., Rogers-Freezer L. (2006). A phase i study on adoptive immunotherapy using gene-modified T cells for ovarian cancer. Clin. Cancer Res..

[24] Lamers C.H., Sleijfer S., van Steenbergen S., van Elzakker P., van Krimpen B., Groot C., Vulto A., den Bakker M., Oosterwijk E., Debets R. (2013). Treatment of metastatic renal cell carcinoma with caix car-engineered T cells: Clinical evaluation and management of on-target toxicity. Mol. Ther..

[25] Savoldo B., Ramos C.A., Liu E., Mims M.P., Keating M.J., Carrum G., Kamble R.T., Bollard C.M., Gee A.P., Mei Z. (2011). Cd28 costimulation improves expansion and persistence of chimeric antigen receptor-modified T cells in lymphoma patients. J. Clin. Invest..

[26] Pule M.A., Savoldo B., Myers G.D., Rossig C., Russell H.V., Dotti G., Huls M.H., Liu E., Gee A.P., Mei Z. (2008). Virus-specific T cells engineered to coexpress tumor-specific receptors: Persistence and antitumor activity in individuals with neuroblastoma. Nat. Med..

[27] Louis C.U., Savoldo B., Dotti G., Pule M., Yvon E., Myers G.D., Rossig C., Russell H.V., Diouf O., Liu E. (2011). Antitumor activity and long-term fate of chimeric antigen receptor-positive T cells in patients with neuroblastoma. Blood.

[28] Sadelain M., Brentjens R., Riviere I. (2013). The basic principles of chimeric antigen receptor design. Cancer Discov..

[29] Kloss C.C., Condomines M., Cartellieri M., Bachmann M., Sadelain M. (2013). Combinatorial antigen recognition with balanced signaling promotes selective tumor eradication by engineered T cells. Nat. Biotechnol..

[30] Lanitis E., Poussin M., Klattenhoff A.W., Song D., Sandaltzopoulos R., June C.H., Powell D.J. (2013). Chimeric antigen receptor T cells with dissociated signaling domains exhibit focused antitumor activity with reduced potential for toxicity *in vivo*. Cancer Immunol. Res..

[31] Wilkie S., van Schalkwyk M.C., Hobbs S., Davies D.M., van der Stegen S.J., Pereira A.C., Burbridge S.E., Box C., Eccles S.A., Maher J. (2012). Dual targeting of erbb2 and muc1 in breast cancer using chimeric antigen receptors engineered to provide complementary signaling. J. Clin. Immunol..

[32] Grada Z., Hegde M., Byrd T., Shaffer D.R., Ghazi A., Brawley V.S., Corder A., Schonfeld K., Koch J., Dotti G. (2013). Tancar: A novel bispecific chimeric antigen receptor for cancer immunotherapy. Mol. Ther. Nucleic Acids.

[33] Hombach A., Sent D., Schneider C., Heuser C., Koch D., Pohl C., Seliger B., Abken H. (2001). T-cell activation by recombinant receptors: Cd28 costimulation is required for interleukin 2 secretion and receptor-mediated t-cell proliferation but does not affect receptor-mediated target cell lysis. Cancer Res..

[34] Imai C., Mihara K., Andreansky M., Nicholson I.C., Pui C.H., Geiger T.L., Campana D. (2004). Chimeric receptors with 4–1bb signaling capacity provoke potent cytotoxicity against acute lymphoblastic leukemia. Leukemia.

[35] Zhang H., Snyder K.M., Suhoski M.M., Maus M.V., Kapoor V., June C.H., Mackall C.L. (2007). 4–1bb is superior to cd28 costimulation for generating cd8+ cytotoxic lymphocytes for adoptive immunotherapy. J. Immunol..

[36] Gong M.C., Latouche J.B., Krause A., Heston W.D., Bander N.H., Sadelain M. (1999). Cancer patient T cells genetically targeted to prostate-specific membrane antigen specifically lyse prostate cancer cells and release cytokines in response to prostate-specific membrane antigen. Neoplasia.

[37] Maher J., Brentjens R.J., Gunset G., Riviere I., Sadelain M. (2002). Human t-lymphocyte cytotoxicity and proliferation directed by a single chimeric tcrzeta /cd28 receptor. Nat. Biotech..

[38] Jensen M.C., Popplewell L., Cooper L.J., DiGiusto D., Kalos M., Ostberg J.R., Forman S.J. (2010). Antitransgene rejection responses contribute to attenuated persistence of adoptively transferred CD20/CD19-specific chimeric antigen receptor redirected T cells in humans. Biol. Blood Marrow Transpl..

[39] Till B.G., Jensen M.C., Wang J., Chen E.Y., Wood B.L., Greisman H.A., Qian X., James S.E., Raubitschek A., Forman S.J. (2008). Adoptive immunotherapy for indolent non-hodgkin lymphoma and mantle cell lymphoma using genetically modified autologous CD20-specific T cells. Blood.

[40] Till B.G., Jensen M.C., Wang J., Qian X., Gopal A.K., Maloney D.G., Lindgren C.G., Lin Y., Pagel J.M., Budde L.E. (2012). CD20-specific adoptive immunotherapy for lymphoma using a chimeric antigen receptor with both cd28 and 4–1bb domains: Pilot clinical trial results. Blood.

[41] Moon E.K., Wang L.C., Dolfi D.V., Wilson C.B., Ranganathan R., Sun J., Kapoor V., Scholler J., Pure E., Milone M.C. (2014). Multifactorial t-cell hypofunction that is reversible can limit the efficacy of chimeric antigen receptor-transduced human T cells in solid tumors. Clin. Cancer Res..

[42] Lamers C.H., Sleijfer S., Vulto A.G., Kruit W.H., Kliffen M., Debets R., Gratama J.W., Stoter G., Oosterwijk E. (2006). Treatment of metastatic renal cell carcinoma with autologous T-lymphocytes genetically retargeted against carbonic anhydrase ix: First clinical experience. J. Clin. Oncol..

[43] Carpenito C., Milone M.C., Hassan R., Simonet J.C., Lakhal M., Suhoski M.M., Varela-Rohena A., Haines K.M., Heitjan D.F., Albelda S.M. (2009). Control of large, established tumor xenografts with genetically retargeted human T cells containing CD28 and CD137 domains. Proc. Natl. Acad. Sci. USA.

[44] Beatty G.L., Haas A.R., Maus M.V., Torigian D.A., Soulen M.C., Plesa G., Chew A., Zhao Y., Levine B.L., Albelda S.M. (2014). Mesothelin-specific chimeric antigen receptor mRNA-engineered T cells induce anti-tumor activity in solid malignancies. Cancer Immunol. Res..

[45] Maus M.V., Haas A.R., Beatty G.L., Albelda S.M., Levine B.L., Liu X., Zhao Y., Kalos M., June C.H. (2013). T cells expressing chimeric antigen receptors can cause anaphylaxis in humans. Cancer Immunol. Res..

[46] Zhao Y., Moon E., Carpenito C., Paulos C.M., Liu X., Brennan A.L., Chew A., Carroll R.G., Scholler J., Levine B.L. (2010). Multiple injections of electroporated autologous T cells expressing a chimeric antigen receptor mediate regression of human disseminated tumor. Cancer Res..

[47] Hinrichs C.S., Borman Z.A., Gattinoni L., Yu Z., Burns W.R., Huang J., Klebanoff C.A., Johnson L.A., Kerkar S.P., Yang S. (2011). Human effector cd8+ T cells derived from naive rather than memory subsets possess superior traits for adoptive immunotherapy. Blood.

[48] Zhang Y., Joe G., Hexner E., Zhu J., Emerson S.G. (2005). Host-reactive cd8+ memory stem cells in graft-versus-host disease. Nat. Med..

[49] Berger C., Jensen M.C., Lansdorp P.M., Gough M., Elliott C., Riddell S.R. (2008). Adoptive transfer of effector CD8+ T cells derived from central memory cells establishes persistent t cell memory in primates. J. Clin. Invest..

[50] Cieri N., Camisa B., Cocchiarella F., Forcato M., Oliveira G., Provasi E., Bondanza A., Bordignon C., Peccatori J., Ciceri F. (2013). Il-7 and il-15 instruct the generation of human memory stem T cells from naive precursors. Blood.

[51] Chinnasamy D., Yu Z., Theoret M.R., Zhao Y., Shrimali R.K., Morgan R.A., Feldman S.A., Restifo N.P., Rosenberg S.A. (2010). Gene therapy using genetically modified lymphocytes targeting vegfr-2 inhibits the growth of vascularized syngenic tumors in mice. J. Clin. Invest..

[52] Davila M.L., Kloss C.C., Gunset G., Sadelain M. (2013). Cd19 car-targeted T cells induce long-term remission and B cell aplasia in an immunocompetent mouse model of B cell acute lymphoblastic leukemia. PloS ONE.

[53] Berger C., Sommermeyer D., Hudecek M., Berger M., Balakrishnan A., Paszkiewicz P.J., Kosasih P.L., Rader C., Riddell S.R. (2015). Safety of targeting ror1 in primates with chimeric antigen receptor-modified T cells. Cancer Immunol. Res..

[54] Dutour A., Marin V., Pizzitola I., Valsesia-Wittmann S., Lee D., Yvon E., Finney H., Lawson A., Brenner M., Biondi A. (2012). *In vitro* and *in vivo* antitumor effect of anti-CD33 chimeric receptor-expressing ebv-ctl against cd33 acute myeloid leukemia. Adv. Hematol..

[55] Casucci M., Nicolis di Robilant B., Falcone L., Camisa B., Norelli M., Genovese P., Gentner B., Gullotta F., Ponzoni M., Bernardi M. (2013). Cd44v6-targeted T cells mediate potent antitumor effects against acute myeloid leukemia and multiple myeloma. Blood.

[56] Mardiros A., Dos Santos C., McDonald T., Brown C.E., Wang X., Budde L.E., Hoffman L., Aguilar B., Chang W.C., Bretzlaff W. (2013). T cells expressing CD123-specific chimeric antigen receptors exhibit specific cytolytic effector functions and antitumor effects against human acute myeloid leukemia. Blood.

[57] Gill S., Tasian S.K., Ruella M., Shestova O., Li Y., Porter D.L., Carroll M., Danet-Desnoyers G., Scholler J., Grupp S.A. (2014). Preclinical targeting of human acute myeloid leukemia and myeloablation using chimeric antigen receptor-modified T cells. Blood.

[58] Fedorov V.D., Themeli M., Sadelain M. (2013). Pd-1- and ctla-4-based inhibitory chimeric antigen receptors (icars) divert off-target immunotherapy responses. Sci. Transl. Med..

[59] Howe S.J., Mansour M.R., Schwarzwaelder K., Bartholomae C., Hubank M., Kempski H., Brugman M.H., Pike-Overzet K., Chatters S.J., de Ridder D. (2008). Insertional mutagenesis combined with acquired somatic mutations causes leukemogenesis following gene therapy of scid-x1 patients. J. Clin. Invest..

[60] Scholler J., Brady T.L., Binder-Scholl G., Hwang W.T., Plesa G., Hege K.M., Vogel A.N., Kalos M., Riley J.L., Deeks S.G. (2012). Decade-long safety and function of retroviral-modified chimeric antigen receptor T cells. Sci. Transl. Med..

[61] Ciceri F., Bonini C., Marktel S., Zappone E., Servida P., Bernardi M., Pescarollo A., Bondanza A., Peccatori J., Rossini S. (2007). Antitumor effects of hsv-tk-engineered donor lymphocytes after allogeneic stem-cell transplantation. Blood.

[62] Ciceri F., Bonini C., Stanghellini M.T.L., Bondanza A., Traversari C., Salomoni M., Turchetto L., Colombi S., Bernardi M., Peccatori J. (2009). Infusion of suicide-gene-engineered donor lymphocytes after family haploidentical haemopoietic stem-cell transplantation for leukaemia (the tk007 trial): A non-randomised phase I-II study. Lancet Oncol..

[63] Tiraby M., Cazaux C., Baron M., Drocourt D., Reynes J.P., Tiraby G. (1998). Concomitant expression of *E. Coli* cytosine deaminase and uracil phosphoribosyltransferase improves the cytotoxicity of 5-fluorocytosine. FEMS Microbiol. Lett..

[64] Di Stasi A., Tey S.K., Dotti G., Fujita Y., Kennedy-Nasser A., Martinez C., Straathof K., Liu E., Durett A.G., Grilley B. (2011). Inducible apoptosis as a safety switch for adoptive cell therapy. New Engl. J. Med..

[65] Clackson T., Yang W., Rozamus L.W., Hatada M., Amara J.F., Rollins C.T., Stevenson L.F., Magari S.R., Wood S.A., Courage N.L. (1998). Redesigning an fkbp-ligand interface to generate chemical dimerizers with novel specificity. Proc. Natl. Acad. Sci. USA.

[66] Griffioen M., van Egmond E.H., Kester M.G., Willemze R., Falkenburg J.H., Heemskerk M.H. (2009). Retroviral transfer of human CD20 as a suicide gene for adoptive t-cell therapy. Haematologica.

[67] Introna M., Barbui A.M., Bambacioni F., Casati C., Gaipa G., Borleri G., Bernasconi S., Barbui T., Golay J., Biondi A. (2000). Genetic modification of human T cells with cd20: A strategy to purify and lyse transduced cells with anti-CD20 antibodies. Human Gene Ther..

[68] Serafini M., Manganini M., Borleri G., Bonamino M., Imberti L., Biondi A., Golay J., Rambaldi A., Introna M. (2004). Characterization of CD20-transduced t lymphocytes as an alternative suicide gene therapy approach for the treatment of graft-versus-host disease. Human Gene Ther..

[69] Wang X., Chang W.C., Wong C.W., Colcher D., Sherman M., Ostberg J.R., Forman S.J., Riddell S.R., Jensen M.C. (2011). A transgene-encoded cell surface polypeptide for selection, *in vivo* tracking, and ablation of engineered cells. Blood.

[70] Kieback E., Charo J., Sommermeyer D., Blankenstein T., Uckert W. (2008). A safeguard eliminates T cell receptor gene-modified autoreactive T cells after adoptive transfer. Proc. Natl .Acad. Sci. USA.

[71] Bonini C., Ferrari G., Verzeletti S., Servida P., Zappone E., Ruggieri L., Ponzoni M., Rossini S., Mavilio F., Traversari C. (1997). Hsv-tk gene transfer into donor lymphocytes for control of allogeneic graft-versus-leukemia. Science.

[72] Tiberghien P., Ferrand C., Lioure B., Milpied N., Angonin R., Deconinck E., Certoux J.M., Robinet E., Saas P., Petracca B. (2001). Administration of herpes simplex-thymidine kinase-expressing donor T cells with a t-cell-depleted allogeneic marrow graft. Blood.

[73] Zhou X., Di Stasi A., Tey S.K., Krance R.A., Martinez C., Leung K.S., Durett A.G., Wu M.F., Liu H., Leen A.M. (2014). Long-term outcome after haploidentical stem cell transplant and infusion of T cells expressing the inducible caspase 9 safety transgene. Blood.

[74] Philip B., Kokalaki E., Mekkaoui L., Thomas S., Straathof K., Flutter B., Marin V., Marafioti T., Chakraverty R., Linch D. (2014). A highly compact epitope-based marker/suicide gene for easier and safer T-cell therapy. Blood.

[75] Moolten F.L. (1986). Tumor chemosensitivity conferred by inserted herpes thymidine kinase genes: Paradigm for a prospective cancer control strategy. Cancer Res..

[76] Beltinger C., Fulda S., Kammertoens T., Meyer E., Uckert W., Debatin K.M. (1999). Herpes simplex virus thymidine kinase/ganciclovir-induced apoptosis involves ligand-independent death receptor aggregation and activation of caspases. Proc. Natl. Acad. Sci. USA.

[77] Spencer D.M., Wandless T.J., Schreiber S.L., Crabtree G.R. (1993). Controlling signal transduction with synthetic ligands. Science.

[78] Iuliucci J.D., Oliver S.D., Morley S., Ward C., Ward J., Dalgarno D., Clackson T., Berger H.J. (2001). Intravenous safety and pharmacokinetics of a novel dimerizer drug, ap1903, in healthy volunteers. J. Clin. Pharmacol..

[79] Oliveira G., Greco R., Lupo-Stanghellini M.T., Vago L., Bonini C. (2012). Use of tk-cells in haploidentical hematopoietic stem cell transplantation. Curr. Opin. Hematol..

[80] Vago L., Perna S.K., Zanussi M., Mazzi B., Barlassina C., Stanghellini M.T., Perrelli N.F., Cosentino C., Torri F., Angius A. (2009). Loss of mismatched hla in leukemia after stem-cell transplantation. New Engl. J. Med..

[81] Traversari C., Marktel S., Magnani Z., Mangia P., Russo V., Ciceri F., Bonini C., Bordignon C. (2007). The potential immunogenicity of the tk suicide gene does not prevent full clinical benefit associated with the use of tk-transduced donor lymphocytes in hsct for hematologic malignancies. Blood.

[82] Hoyos V., Savoldo B., Quintarelli C., Mahendravada A., Zhang M., Vera J., Heslop H.E., Rooney C.M., Brenner M.K., Dotti G. (2010). Engineering cd19-specific t lymphocytes with interleukin-15 and a suicide gene to enhance their anti-lymphoma/leukemia effects and safety. Leukemia.

[83] Budde L.E., Berger C., Lin Y., Wang J., Lin X., Frayo S.E., Brouns S.A., Spencer D.M., Till B.G., Jensen M.C. (2013). Combining a cd20 chimeric antigen receptor and an inducible caspase 9 suicide switch to improve the efficacy and safety of t cell adoptive immunotherapy for lymphoma. PloS ONE.

[84] Cornelissen J.J., Gratwohl A., Schlenk R.F., Sierra J., Bornhauser M., Juliusson G., Racil Z., Rowe J.M., Russell N., Mohty M. (2012). The european leukemianet aml working party consensus statement on allogeneic hsct for patients with aml in remission: An integrated-risk adapted approach. Nat. Rev. Clin. Oncol..

[85] Amrolia P.J., Muccioli-Casadei G., Yvon E., Huls H., Sili U., Wieder E.D., Bollard C., Michalek J., Ghetie V., Heslop H.E. (2003). Selective depletion of donor alloreactive T cells without loss of antiviral or antileukemic responses. Blood.

[86] Melenhorst J.J., Leen A.M., Bollard C.M., Quigley M.F., Price D.A., Rooney C.M., Brenner M.K., Barrett A.J., Heslop H.E. (2010). Allogeneic virus-specific T cells with hla alloreactivity do not produce gvhd in human subjects. Blood.

[87] Van Loenen M.M., de Boer R., van Liempt E., Meij P., Jedema I., Falkenburg J.H., Heemskerk M.H. (2014). A good manufacturing practice procedure to engineer donor virus-specific T cells into potent anti-leukemic effector cells. Haematologica.

[88] Cruz C.R., Micklethwaite K.P., Savoldo B., Ramos C.A., Lam S., Ku S., Diouf O., Liu E., Barrett A.J., Ito S. (2013). Infusion of donor-derived cd19-redirected virus-specific T cells for B-cell malignancies relapsed after allogeneic stem cell transplant: A phase 1 study. Blood.

[89] Rubio M.T., Moreira-Teixeira L., Bachy E., Bouillie M., Milpied P., Coman T., Suarez F., Marcais A., Sibon D., Buzyn A. (2012). Early posttransplantation donor-derived invariant natural killer t-cell recovery predicts the occurrence of acute graft-versus-host disease and overall survival. Blood.

[90] Lamb L.S., Lopez R.D. (2005). Gammadelta T cells: A new frontier for immunotherapy?. Biol. Blood Marrow Transpl..

[91] Deniger D.C., Switzer K., Mi T., Maiti S., Hurton L., Singh H., Huls H., Olivares S., Lee D.A., Champlin R.E. (2013). Bispecific t-cells expressing polyclonal repertoire of endogenous gammadelta t-cell receptors and introduced cd19-specific chimeric antigen receptor. Mol. Ther..

[92] Provasi E., Genovese P., Lombardo A., Magnani Z., Liu P.Q., Reik A., Chu V., Paschon D.E., Zhang L., Kuball J. (2012). Editing t cell specificity towards leukemia by zinc finger nucleases and lentiviral gene transfer. Nat. Med..

